# Autophagic Activation and Decrease of Plasma Membrane Cholesterol Contribute to Anticancer Activities in Non-Small Cell Lung Cancer

**DOI:** 10.3390/molecules26195967

**Published:** 2021-10-01

**Authors:** Jui-Ling Hsu, Wohn-Jenn Leu, Nan-Shan Zhong, Jih-Hwa Guh

**Affiliations:** 1 School of Pharmacy, National Taiwan University, No. 33, Linsen S. Rd., Zhongzheng Dist., Taipei 100, Taiwan; d97423004@ntu.edu.tw (J.-L.H.); r00423018@gmail.com (W.-J.L.); 2 Department of Pharmacy, New Taipei Municipal TuCheng Hospital, Chang Gung Memorial Hospital, New Taipei City 236, Taiwan; 3 State Key Laboratory of Respiratory Disease, National Clinical Research Center for Respiratory Disease, Guangzhou Institute for Respiratory Health, First Affiliated Hospital of Guangzhou Medical University, Guangzhou 510370, China

**Keywords:** *para*-toluenesulfonamide, Akt/mTOR/p70S6K pathway, plasma membrane cholesterol, autophagy, non-small cell lung cancer

## Abstract

Non-small cell lung cancer (NSCLC), an aggressive subtype of pulmonary carcinomas with high mortality, accounts for 85% of all lung cancers. Drug resistance and high recurrence rates impede the chemotherapeutic effect, making it urgent to develop new anti-NSCLC agents. Recently, we have demonstrated that *para*-toluenesulfonamide is a potential anti-tumor agent in human castration-resistant prostate cancer (CRPC) through inhibition of Akt/mTOR/p70S6 kinase pathway and lipid raft disruption. In the current study, we further addressed the critical role of cholesterol-enriched membrane microdomain and autophagic activation to *para*-toluenesulfonamide action in killing NSCLC. Similar in CRPC, *para*-toluenesulfonamide inhibited the Akt/mTOR/p70S6K pathway in NSCLC cell lines NCI-H460 and A549, leading to G1 arrest of the cell cycle and apoptosis. *Para*-toluenesulfonamide significantly decreased the cholesterol levels of plasma membrane. External cholesterol supplement rescued *para*-toluenesulfonamide-mediated effects. *Para*-toluenesulfonamide induced a profound increase of LC3-II protein expression and a significant decrease of p62 expression. Double staining of lysosomes and cellular cholesterol showed *para*-toluenesulfonamide-induced lysosomal transportation of cholesterol, which was validated using flow cytometric analysis of lysosome staining. Moreover, autophagy inhibitors could blunt *para*-toluenesulfonamide-induced effect, indicating autophagy induction. In conclusion, the data suggest that *para*-toluenesulfonamide is an effective anticancer agent against NSCLC through G1 checkpoint arrest and apoptotic cell death. The disturbance of membrane cholesterol levels and autophagic activation may play a crucial role to *para*-toluenesulfonamide action.

## 1. Introduction

Lung cancer is the leading cause of cancer mortality. Non-small cell lung cancer (NSCLC), which accounts for about 85% of all lung cancers, includes any type of epithelial lung cancer other than small cell lung cancer [1]. There are several subtypes of NSCLC categorized by the origin of different types of lung cells, including adenocarcinoma, squamous cell (epidermoid) carcinoma, large cell (undifferentiated) carcinoma and other types. Among them, adenocarcinoma and squamous cell carcinoma are the two predominant histological phenotypes in NSCLC. Chemotherapy, most often a combination of chemo drugs, is one of the options for advanced cancers or for some patients who are not healthy enough for surgery. However, side effects are inevitable depending on the type and dose of the given drugs as well as the treatment duration [2,3]. Target therapy is another important option for NSCLC treatment, including the targets on angiogenesis or on cancer cells with epidermal growth factor receptor (EGFR) gene mutations, anaplastic lymphoma kinase gene changes, ROS1 fusion and BRAF gene changes. However, most patients inevitably develop acquired resistance to the target therapeutic drugs [4,5]. Therefore, new therapeutic approaches need to be developed to overcome the challenges.

Accumulating evidence reveals that several signals for cell proliferation and survival are transmitted through lipid rafts, specialized membrane microdomains enriched with cholesterol [6]. Epidemiological studies which elucidate the relationship between cholesterol intake and cancer incidence reveal that high cholesterol levels augment the risk of cancers including lung, breast, stomach, colon, pancreas and many other cancers [7,8]. A variety of studies also support that cholesterol homeostasis genes can regulate cancer development [9]. Cells obtain cholesterol from circulation and from de novo synthesis through the mevalonate/isoprenoid/cholesterol pathway in which squalene synthase is a key enzyme in determining the pathway toward cholesterol synthesis [10]. Yang and colleagues have reported that squalene synthase is overexpressed in highly invasive NSCLC and have proposed a potential strategy targeting squalene synthase and cholesterol for NSCLC treatment [11]. Phosphatidylinositol 3-kinase (PI3K)/Akt activation secondary to a mutation in the K-Ras gene or EGFR gene in NSCLC is a key survival signaling pathway. Statins, inhibitors of 3-hydroxy-3-methylglutaryl coenzyme A (HMG-CoA) reductase in cholesterol synthesis, have been suggested to enhance anti-NSCLC effects induced by several anti-tumor agents in which the inhibition of both PI3K/Akt and mitogen-activated protein kinase (MAPK) pathways explain the statins’ sensitization activity [12]. Collectively, these studies suggest a potential role of disturbed cholesterol homeostasis in cancer development. 

*Para*-toluenesulfonamide is a simple small molecule displaying both in vitro and in vivo anticancer activities in a variety of cancers including lung cancer, prostate cancer and tongue squamous cell carcinoma [13,14,15]. The clinical trials show that *para*-toluenesulfonamide displays efficient anti-tumor activity against advanced hepatocellular carcinoma and non-small cell lung cancer through a concurrent local injection therapy [16,17,18,19]. Recently, we have demonstrated that *para*-toluenesulfonamide is a potential anti-tumor agent which inhibits both the Akt-dependent and -independent mammalian target of rapamycin (mTOR)/p70S6 kinase (p70S6K) pathways in prostate cancers, and the disturbance of lipid raft and cholesterol contents also are involved in the *para*-toluenesulfonamide-mediated mechanism [15]. In the present work, we have documented the anticancer effect of *para*-toluenesulfonamide in NSCLC, elucidating the cholesterol action on Akt/mTOR/p70S6K pathways. *Para*-toluenesulfonamide-mediated changes of the plasma membrane cholesterol levels, lysosomal cholesterol levels and autophagic induction also have been studied to understand the *para*-toluenesulfonamide-mediated anti-NSCLC mechanism.

## 2. Results

### 2.1. Para-Toluenesulfonamide Induces Anti-NSCLC Effects through Inhibition of Akt/mTOR/p70S6K Pathway

**Figure 1:**
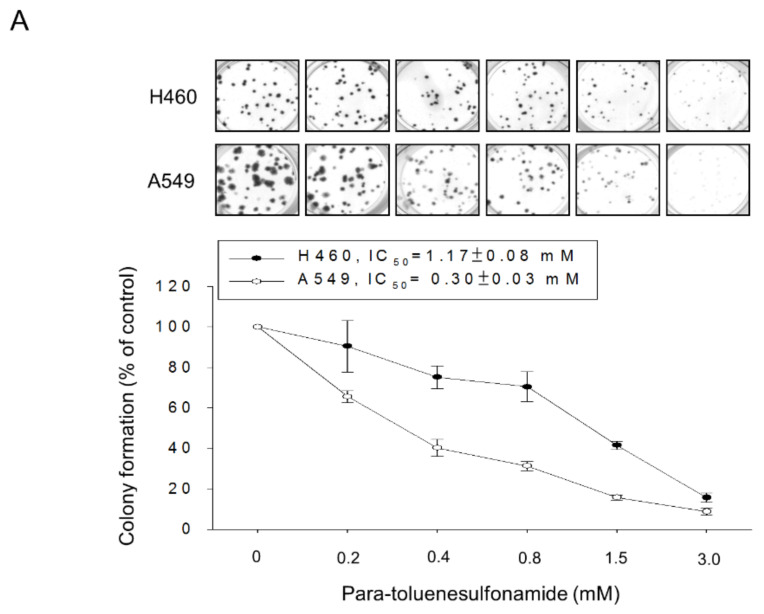

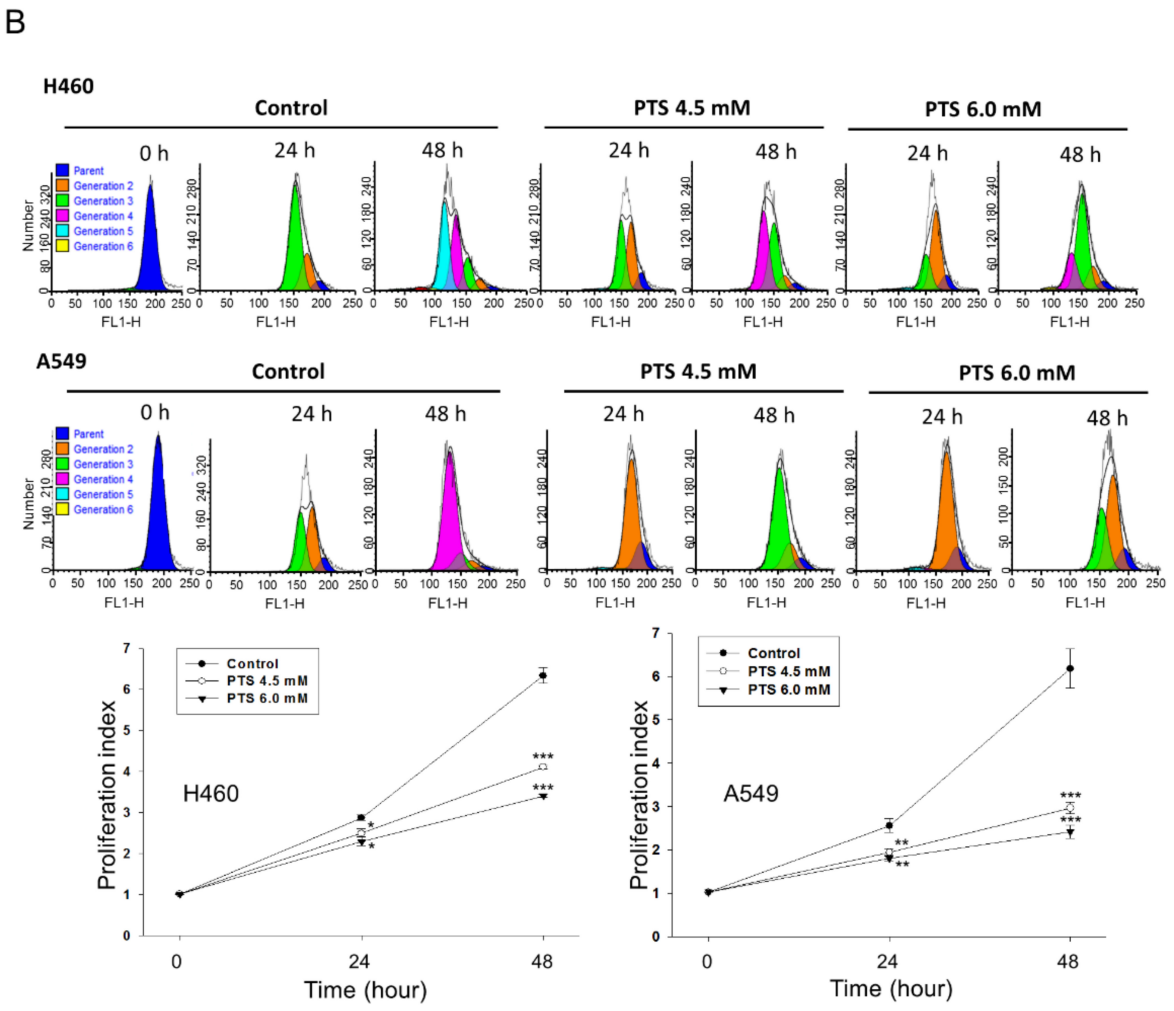
Effect of *para*-toluenesulfonamide on cell proliferation in NCI-H460 and A549 cells. (**A**) The cells were incubated in the absence or presence of *para*-toluenesulfonamide for 10 days. After treatment, cells were fixed and stained for colony formation assay. (**B**) The cells were incubated with or without *para*-toluenesulfonamide. After treatment, cells were harvested for flow cytometric analysis of CFSE staining. The cell populations of parent or different generations and proliferation index were calculated by Modfit LT Version 3.2 and WinList Version 5.0 software. Quantitative data are expressed as mean ± SEM of three independent experiments. * *p* < 0.05, ** *p* < 0.01 and *** *p* < 0.001 compared with the control.

**Figure 2:**
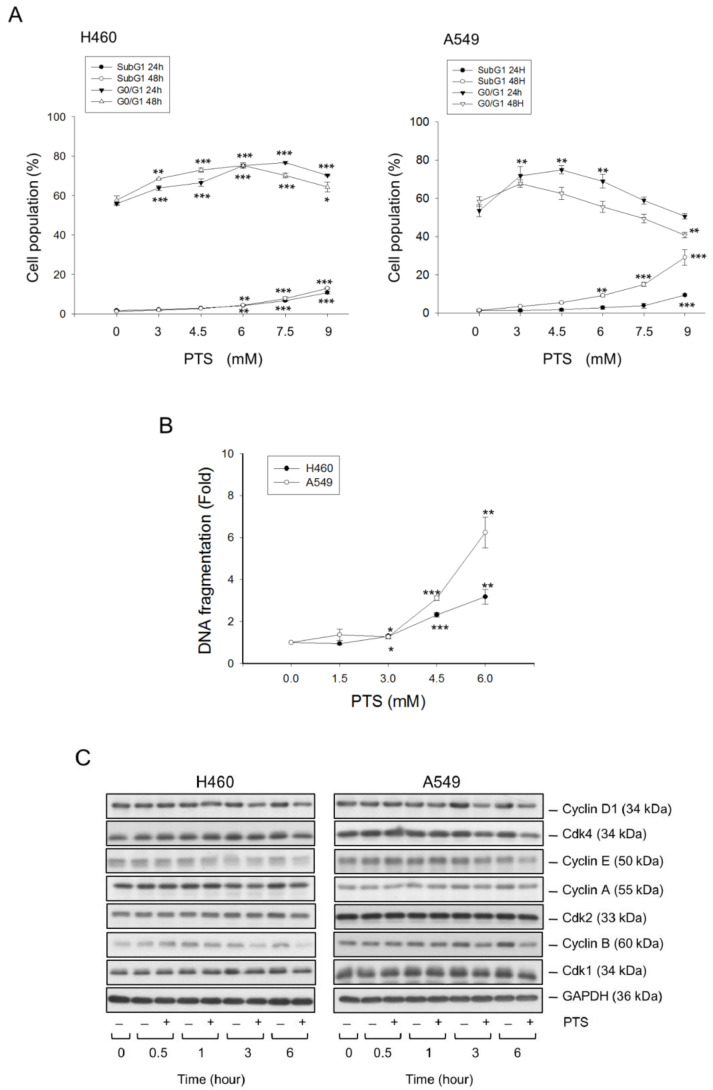
Effect of *para*-toluenesulfonamide on the distribution of cell cycle phases in NCI-H460 and A549 cells. The cells were incubated in the absence or presence of *para*-toluenesulfonamide at the indicated condition (**A**, 24 and 48 h; **B**, 48 h; **C**, 6 mM). After the treatment, the cells were harvested for the detection of cell population at different phases by flow cytometric analysis of PI staining (**A**), the cell apoptosis by detecting nucleosomal DNA fragmentation using Cell Death Detection ELISA^PLUS^ kit (**B**) and the detection of protein expressions of cell cycle regulators by Western blot analysis (**C**). Quantitative data are expressed as mean ± SEM of three independent experiments. * *p* < 0.05, ** *p* < 0.01 and *** *p* < 0.001 compared with the control.

The anti-proliferative activities of *para*-toluenesulfonamide were examined using several assessments including colony formation assay, flow cytometric analysis of CFSE staining and sulforhodamine B assay. All assessments showed that *para*-toluenesulfonamide caused a profound inhibition of cell proliferation in both NCI-H460 and A549 cell lines (Figure 1 and Figure S1 (Supplementary Materials)). Since cells are vulnerable to stress that results in checkpoint arrest of the cell cycle and halts proliferation, we have determined the distribution of cell cycle phases in the study. The flow cytometric analysis of propidium iodide (PI) staining showed that *para*-toluenesulfonamide induced the G1 arrest in both NCI-H460 and A549 cell lines and subsequent apoptosis evident by an increased sub-G1 (apoptosis) population (Figure 2A). Apoptotic cell death was verified by a nucleosomal DNA fragmentation assay based on the quantitation of cytoplasmic histone-associated DNA fragments in cells (Figure 2B). In support of the disturbance of the G1 phase, we examined the protein expressions of various cyclins and Cdks, in particular, the cyclin D1 which was synthesized during the G1 phase and drove the G1-S phase transition. The Western blotting analysis showed that *para*-toluenesulfonamide profoundly inhibited cyclin D1 expression (NCI-H460: 64.8 ± 6.6% and 73.1 ± 4.2% at 3- and 6-h treatment, respectively, vs. 100% of control; A549: 68.9 ± 7.7% and 73.9 ± 5.5% at 3- and 6-h treatment, respectively, vs. 100% of control, all *p* < 0.05, *n* = 3) (Figure 2C).

The regulation of Akt/mTOR/p70S6K axis signaling pathway has been considered to be a potential strategy in anticancer research [20,21,22,23]. Our previous data showed that *para*-toluenesulfonamide induced an inhibitory effect on the phosphorylation and activation of Akt, mTOR and p70S6K in CRPC [15]. We also tested the Akt/mTOR/p70S6K pathway in both NCI-H460 and A549 cell lines. Similar inhibitory effects were obtained in the study. Notably, the suppression of p70S6K activation was the most susceptible to *para*-toluenesulfonamide (Figure S2 (Supplementary Materials)). Furthermore, 4E-BP1 which played a role in inhibiting protein translation through the association with the translation initiation factor eIF4E was influenced in the presence of *para*-toluenesulfonamide, leading to an increase of phosphorylation in the α form of 4E-BP1 but a decrease in the γ form in both NCI-H460 and A549 cell lines (Figure S2 (Supplementary Materials)). Several specific kinase inhibitors were used to establish precisely the participation of these signaling pathways in the observed inhibitory effect. The data showed that MK-2206, a selective inhibitor of Akt, displayed the most effective inhibition on the activities of Akt, mTOR and p70S6K in both NCI-H460 and A549 cell lines. Rapamycin, an mTORC1 inhibitor, inhibited both mTOR and p70S6K; notably, it also partly suppressed the Akt activity, suggesting that mTOR might interactively regulate Akt activity. In contrast, the selective p70S6K inhibitor PF-4708671 had no effect on Akt but inhibited the activities of both mTOR and p70S6K, supporting the reciprocal regulation between these two kinases (Figure S3 (Supplementary Materials)).

### 2.2. Para-Toluenesulfonamide Suppresses Cholesterol Levels of the Plasma Membrane

**Figure 3:**
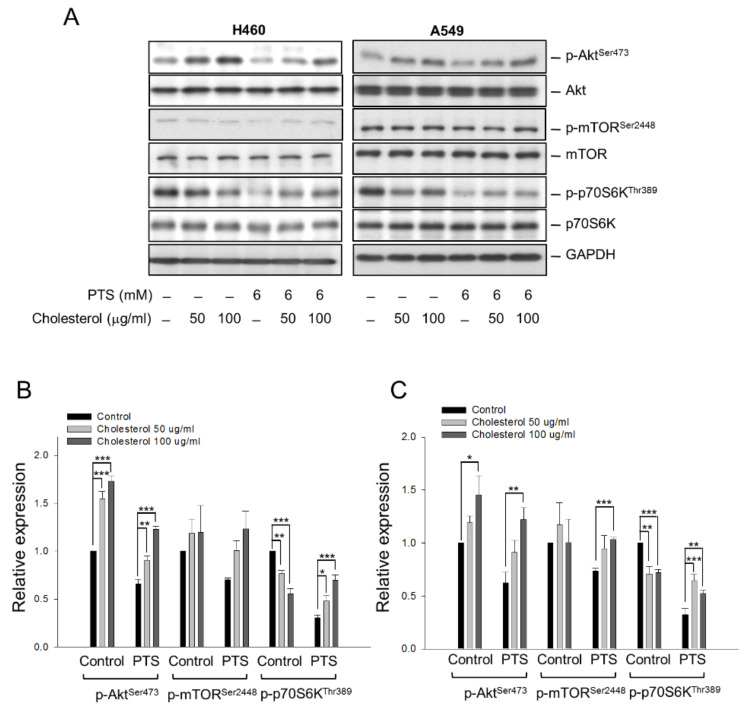
Effect of external cholesterol supplement on *para*-toluenesulfonamide-mediated effects. NCI-H460 and A549 cells were incubated in the absence or presence of the indicated agent for one hour. After the treatment, the cells were harvested and lysed for the detection of protein expressions by Western blot analysis (**A**). The expression was quantified using Image Lab Software 6.0 (BIO-RAD). The protein expression relative to *para*-toluenesulfonamide-free/cholesterol-free control in NCI-H460 (**B**) and A549 cells (**C**) is demonstrated. Data are expressed as mean ± SEM of three determinations. * *p* < 0.05, ** *p* < 0.01 and *** *p* < 0.001 compared with the control.

**Figure 4:**
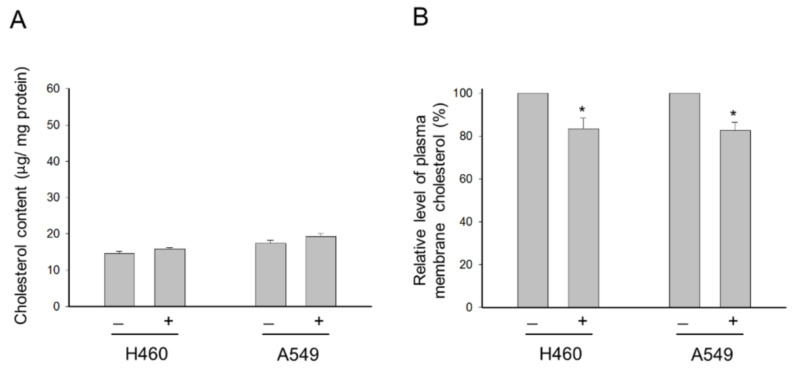
Effect of *para*-toluenesulfonamide on cholesterol content. NCI-H460 and A549 cells were incubated in the absence or presence of 6 mM *para*-toluenesulfonamide for 24 h. (**A**) The level of total cellular cholesterol was examined using the Cholesterol Assay Kit. (**B**) Plasma membrane fraction was isolated by sucrose density gradient centrifugation, and the level of cholesterol on plasma membrane was examined using the Cholesterol Assay Kit. Na^+^/K^+^ ATPase a1-subunit was used as internal control. The data are expressed as mean ± SEM of three independent determinations. * *p* < 0.05 compared with the control.

Cholesterol is a well-identified crucial component of lipid rafts in cells, and membrane cholesterol levels have been suggested to be a central factor in maintaining raft stability and organization [24]. Several studies have revealed that Akt activation and cell survival are regulated by cholesterol-sensitive signaling pathways [25]. In the present work, we have examined the effect of cholesterol on the activities of Akt, mTOR and p70S6K. As a result, external cholesterol supplement, by itself, induced a significant increase in Akt phosphorylation at Ser^473^, indicating increased Akt activity in both NCI-H460 and A549 cells (Figure 3). In contrast, external cholesterol inhibited the phosphorylation and activation of p70S6K (Figure 3). Nevertheless, *para*-toluenesulfonamide-mediated inhibition of Akt, mTOR and p70S6K activities was drastically rescued by the external cholesterol supplement (Figure 3). The data suggest that the disturbance of cellular cholesterol homeostasis may participate in *para*-toluenesulfonamide-mediated signaling pathway. However, further identification showed that *para*-toluenesulfonamide did not change the total cellular cholesterol contents but significantly decreased the cholesterol levels of the plasma membrane in both NCI-H460 and A549 cells (Figure 4).

### 2.3. Lysosome Plays a Role in Determining the Fate of Cholesterol through Autophagic Activation

**Figure 5:**
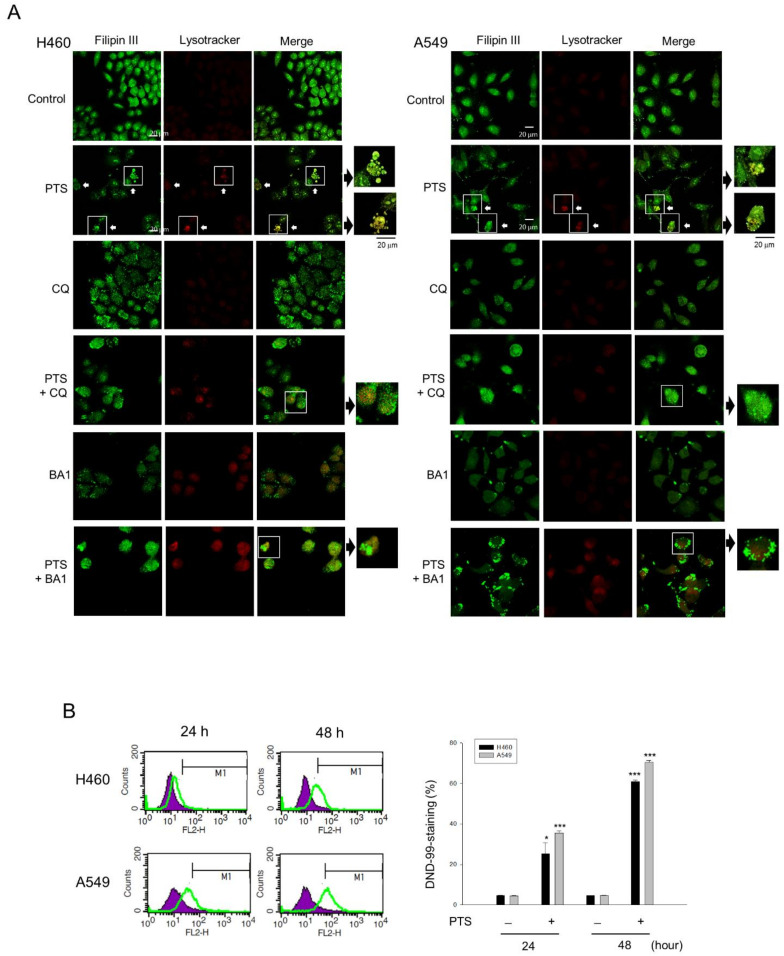
Effect of *para*-toluenesulfonamide on cholesterol distribution. (**A**) NCI-H460 and A549 cells were incubated in the absence or presence of the indicated agent (*para*-toluenesulfonamide, 6 mM; CQ, 50 μM chloroquine; BA1, 0.1 μM bafilomycin A1) for 24 h. Cellular cholesterol distribution was determined by Filipin III staining (green fluorescence) coupled with Lysotracker DND-99 immunofluorescence staining (red fluorescence). The arrow indicates the plasma membrane cholesterol. The yellow fluorescence indicates the co-localization of cholesterol and lysosomes. (**B**) The cells were incubated in the absence or presence of 6 mM PTS for 24 or 48 h. The cells were harvested for the detection of lysosome formation using flow cytometric analysis of LysoTracker Red-DND-99 staining. The data are expressed as mean ± SEM of three independent determinations. * *p* < 0.05 and *** *p* < 0.001 compared with the control.

**Figure 6:**
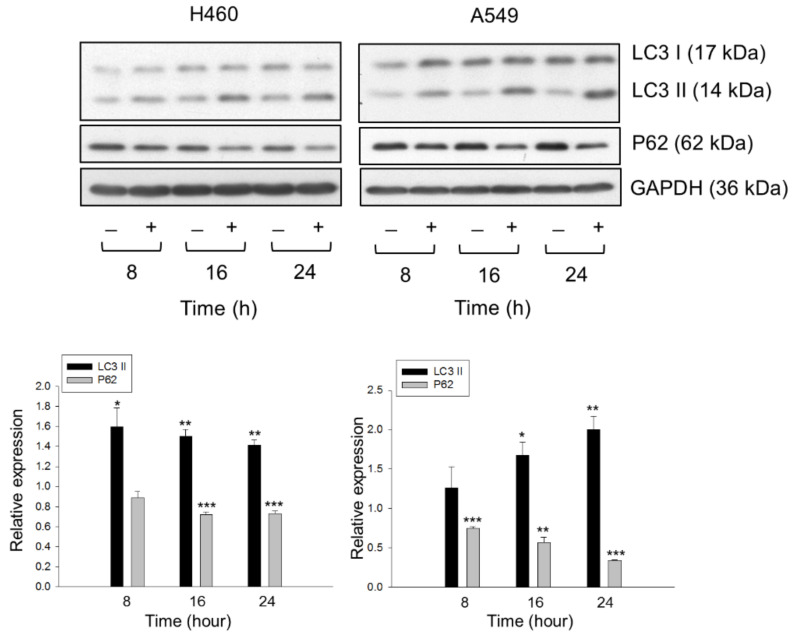
Effect of *para*-toluenesulfonamide on LC3 and p62 protein expressions in NCI-H460 and A549 cells. The cells were incubated in the absence or presence of 6 mM *para*-toluenesulfonamide for the indicated times. After treatment, the cells were harvested and lysed for the detection of protein expressions by Western blot analysis. Protein expressions were quantified using Image Lab Software 6.0 (BIO-RAD). The protein expression relative to respective time control is demonstrated. Data are expressed as mean ± SEM of three determinations. * *p* < 0.05, ** *p* < 0.01 and *** *p* < 0.001 compared with *para*-toluenesulfonamide-free control.

It has been evident that cholesterol depletion in lipid rafts is able to induce both autophagy and apoptosis through the PI3K/Akt pathway [26,27,28]. Since *para*-toluenesulfonamide inhibited Akt/mTOR/p70S6K pathways and reduced the membrane cholesterol levels, the role of lysosome and autophagic determination in the fate of cholesterol was examined. By using the staining with LysoTracker Red DND-99 and cholesterol detector Filipin III, the data showed that exposure of cells to *para*-toluenesulfonamide induced the co-localization of both staining in NCI-H460 and A549 cells, indicating the lysosomal transportation of cholesterol. This effect was inhibited in the presence of chloroquine and bafilomycin A1, two inhibitors of autophagy (Figure 5A). The lysosomal transportation effect was validated and quantitatively measured using flow cytometric analysis of DND-99 staining (Figure 5B). Moreover, further substantiation demonstrated that *para*-toluenesulfonamide resulted in a profound increase in protein expression in LC3-II and a significant decrease in p62 protein levels in both cell lines (Figure 6). The autophagy inhibitors, 3-methyladenine and chloroquine, further increased the protein levels of LC3-II and rescued p62 protein expression in the presence of *para*-toluenesulfonamide in both NCI-H460 and A549 cell lines (Figure S4 (Supplementary Materials)). As the conversion of LC3-I to lipid-bound LC3-II is associated with the formation of autophagosomes, and as p62 is an autophagy substrate used as a detector of autophagy activity, our data provided evidence supporting the induction of autophagy to *para*-toluenesulfonamide action. Furthermore, the autophagy inhibitors chloroquine and bafilomycin A1 significantly increased *para*-toluenesulfonamide-induced sub-G1 population (apoptosis); however, both autophagy inhibitors did not correspondingly augment *para*-toluenesulfonamide-induced cell death using MTT assay (Figure S5 (Supplementary Materials)). The data indicated that *para*-toluenesulfonamide-induced total cell death of all possible forms (e.g., autophagy and apoptosis) would be similar even in the presence of autophagy inhibitor. The data also suggested that despite the presence of protective autophagy against apoptosis, cells enter cell death stages in continuous presence of *para*-toluenesulfonamide, underlining the potential of this compound in anti-NSCLC activity.

### 2.4. Para-Toluenesulfonamide Synergistically Potentiates Gefitinib-Induced Cell Death

**Figure 7:**
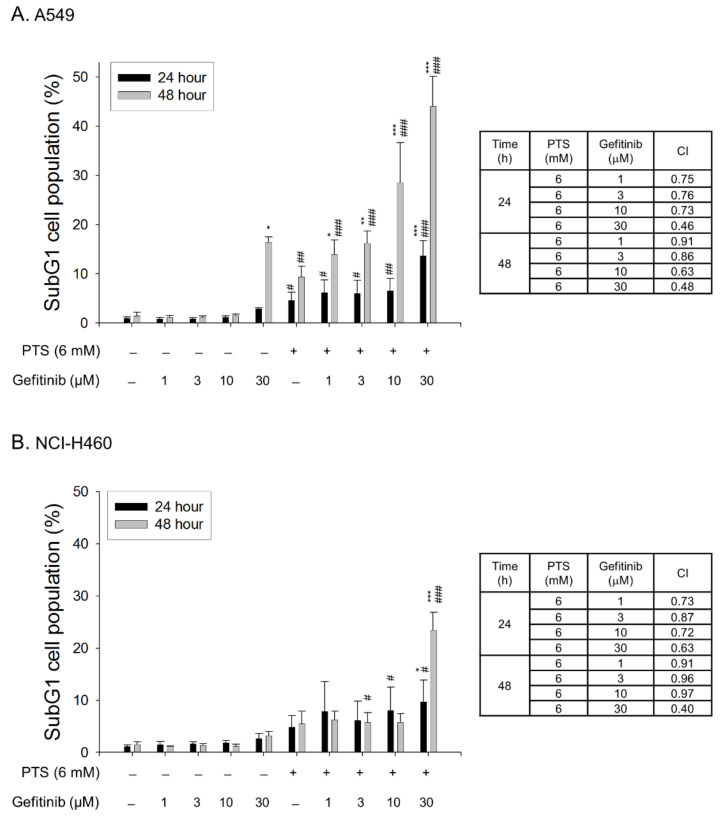
Effect of combination between *para*-toluenesulfonamide and gefitinib on cell death. A549 (**A**) and NCI-H460 cells (**B**) were treated with or without the indicated agent for 24 or 48 h. Then, the cells were obtained for examining cell population at sub-G1 phase by cytoflowmetry experiment of PI staining. The resulting combination index (CI) values were less than 1.0 confirming the synergistic effects. Quantitative data are expressed as mean ± SEM of three independent experiments. * *p* < 0.05, ** *p* < 0.01 and *** *p* < 0.001 compared with the drug-free control. # *p* < 0.05, ## *p* < 0.01 and ### *p* < 0.001 compared with the respective concentration of gefitinib.

The combinatory treatment between *para*-toluenesulfonamide and therapeutic agents in both A549 and NCI-H460 cell lines was performed. The synergism between *para*-toluenesulfonamide and gefitinib was assessed through constructing isobolograms and calculating combination index (CI) values using Chou-Talalay method [29]. The resulting CI values were less than 1.0 confirming the synergistic effects. The data showed that the sub-G1 population (cell death) was synergistically increased in both A549 and NCI-H460 cell lines responsive to the combination between *para*-toluenesulfonamide and gefitinib (Figure 7A,B) but not that with paclitaxel or cisplatin (Figure S6 (Supplementary Materials)).

## 3. Discussion

The roles of cholesterol in regulating cancer growth and development and targeting cholesterol biosynthesis and homeostasis for therapeutic development are an attractive and important area in cancer research. A variety of studies in several main areas, such as epidemiologic studies both in in vitro and in vivo pharmacological and biochemical studies and the Cancer Genome Atlas (TCGA) database studies, provide evidence supporting an association between cancers and cholesterol levels. A meta-analysis of case–control and cohort studies, performed to assess the relationship between lung cancer risk and dietary cholesterol intake, has revealed a positive correlation based on the case–control studies, although the cohort studies show inconsistent results [30]. Several TCGA database studies have profiled the expression levels of thousands of genes involved in cholesterol metabolism in various cancers [9,31]. Moreover, several lines of evidence show that statins may sensitize anticancer effects induced by cancer chemotherapeutic drugs [12,32]. Together, these studies suggest a crucial role of cholesterol in cancer growth and development and support the notion that targeting cholesterol may be a novel therapeutic strategy for cancer treatment [33]. Our previous work has demonstrated that *para*-toluenesulfonamide decreases the phosphorylation of several lipid raft- associated survival kinases in CRPC and has suggested that *para*-toluenesulfonamide may display anticancer activity through a signaling pathway related to disturbance of intracellular cholesterol homeostasis [15]. In the present work, we have elucidated the anticancer mechanism of *para*-toluenesulfonamide in NSCLC in examining the role of plasma membrane cholesterol levels, lysosomal cholesterol levels and autophagic induction.

Several assessments including SRB assay, colony formation assay and CFSE cell proliferation test substantiated the anti-proliferative activities of *para*-toluenesulfonamide in both NSCLC NCI-H460 and A549 cell lines. Of note, *para*-toluenesulfonamide showed higher activity in A549 than in NCI-H460 cells. The data also demonstrated that *para*-toluenesulfonamide displayed higher activity in colony formation assay than in SRB assay. This might be due to the high susceptibility of a single cell to *para*-toluenesulfonamide in colony formation assay. A long-term treatment of ten days could also possibly explain the higher activity of *para*-toluenesulfonamide in the colony formation assay. Moreover, *para*-toluenesulfonamide induced G1 arrest of the cell cycle and subsequent cell apoptosis. Because the G1 phase is of particular importance in synthesizing mRNA and proteins required for DNA synthesis, the induction of G1 arrest may indicate a translational interruption [34]. The Akt/mTOR pathway, which can affect downstream effectors p70S6K and 4E-BP1, has been studied as a central regulator in translational control and cell survival. High levels of phosphorylated Akt and mTOR, which indicate kinase activation, are associated with poor prognosis in patients with NSCLC [35,36]. Furthermore, the levels of both total and phosphorylated p70S6K are profoundly higher in tumors than in normal specimens from NSCLC patients [37]. *Para*-toluenesulfonamide displayed a remarkable inhibition of Akt/mTOR/p70S6K signaling pathway, indicating its anti-NSCLC potential.

eIF4E is an mRNA cap binding protein in regulating eukaryotic translation. In contrast, 4E-BP1 is a key regulator which inhibits protein translation through the association with eIF4E. Therefore, inactivation of 4E-BP1 can lead to eIF4E activation and translation initiation. More specifically, 4E-BP1 consists of several isoforms including α, β and γ isoforms. It has been reported that 4E-BP1 α form plays a key role since its hypophosphorylated or nonphosphorylatable form is extensively ubiquitinated and degraded, leading to the dissociation and activation of eIF4E [38,39]. In contrast, an increase in phosphorylated 4E-BP1 γ form and a subsequent decrease in association between 4E-BP1 and eIF4E have been documented in cells responsive to growth stimulation, such as insulin and insulin-like growth factor-1 [40]. These data suggest a decrease in phosphorylated 4E-BP1 α form, while an increase in phosphorylated 4E-BP1 γ form is crucial for protein translation. Our data showed that *para*-toluenesulfonamide counteracted these effects, leading to a profound increase in phosphorylated 4E-BP1 α form but a decrease in phosphorylated γ form in both NCI-H460 and A549 cell lines, indicating the inhibition of protein translation.

Cell membrane has the highest concentration of cholesterol. Membrane lipid rafts, which are highly ordered membrane domains enriched in cholesterol, may provide a crucial microenvironment serving as key modulators in the signal transduction of immunity, adhesion, metastasis, proliferation and survival [6,11,15,23,25]. The lipid raft platforms may mediate signaling generated from the activation of growth factor receptors (e.g., insulin-like growth factor-1 receptor and vascular endothelial growth factor receptor), serine/threonine kinase (e.g., Akt and mTOR), tyrosine kinase (e.g., c-met and c-Src), death receptor (e.g., Fas/CD95 receptor, DR4 and DR5), cytoskeletal components, integrins and ion channels [6,41,42]. Numerous studies have suggested that Akt activation through the stimulation of growth factor receptors, such as insulin-like growth factor-1 receptor, is dependent on lipid rafts [43]. The synthetic lipids, edelfosine and perifosine, which target lipid rafts [6,44], have been reported to display anti-tumor activity through raft reorganization, leading to the displacing of Akt from lipid rafts. Our data demonstrated that external cholesterol supplement substantially rescued *para*-toluenesulfonamide-mediated inhibition of Akt/mTOR/p70S6K pathway in NSCLC cells, suggesting the possible impact of *para*-toluenesulfonamide on raft organization and cholesterol content. The localization of cellular cholesterol may affect cell survival. Lima and colleagues have reported that a thioxanthone derivative displays anti-NSCLC activity, which is associated with a redistribution of cholesterol from plasma membrane to cytoplasm [45]. Lipid rafts have been identified serving as a player of the autophagic process. The alteration of cellular lipid environment, presented by cellular cholesterol depletion or changed cholesterol trafficking, has been substantiated to induce autophagy [27,45]. The results that *para*-toluenesulfonamide significantly decreased the cholesterol levels of the plasma membrane in both NCI-H460 and A549 cells were similar to those reported by Lima and colleagues [45], and autophagy was induced accordingly. However, *para*-toluenesulfonamide-mediated autophagy induction might also be attributed to the inhibition of Akt and mTOR since both Akt and mTORC1 could inhibit the pro-autophagic function, and conversely, suppression of Akt and mTORC1 induced autophagy [46,47]. Furthermore, it has been documented that when mTORC1 activity is low, cholesterol can be transported to lysosomes through the autophagy-involved membrane trafficking pathway [48]. Therefore, the inhibition of mTOR activity could be responsible for *para*-toluenesulfonamide-induced lysosomal transportation of cholesterol and autophagy. Notably, ruptured plasma membranes are generally considered as hallmarks of necrotic cell death. Necroptosis and pyroptosis are two forms of regulated necrosis, revealing the plasma membrane rupture depending on the polymerization of mixed lineage kinase domain-like pseudokinase (MLKL) and pore formation of non-selective gasdermin D, respectively [49]. Regarding this point, we examined the effect of *para*-toluenesulfonamide on the release reaction of lactate dehydrogenase (LDH), a biomarker of both necroptosis and pyroptosis. However, *para*-toluenesulfonamide did not induced a significant increase of LDH release until the exposure at a high concentration of 7.5 mM that caused 3.9 ± 0.8% and 1.7 ± 0.2% increase of LDH release in A549 and NCI-H460 cells, respectively (*n* = 3). The data suggested that *para*-toluenesulfonamide did not cause massive necrotic cell death and implied that *para*-toluenesulfonamide influenced the plasma membrane cholesterol possibly not through the plasma membrane rupture.

Aside from the cell membrane, the synthesized cholesterol in the endoplasmic reticulum (ER) can also be transported to numerous organelles, such as mitochondria and Golgi membranes. The biogenesis, maintenance of membrane and steroid hormone biosynthesis in the mitochondria needs the involvement of cholesterol. Steroid synthesis in steroidogenic cells is started at the inner mitochondrial membrane, where cholesterol side-chain cleavage enzyme CYP11A1 triggers the conversion of cholesterol to pregnenolone, which enters the ER for further enzymatic reactions [50,51]. Several in vitro mitochondria models in which the mitochondria rich in cholesterol or isolated mitochondria from cells with aberrant mitochondrial cholesterol gathering provide evidence supporting that the levels of mitochondrial cholesterol may impact on mitochondrial function [52]. Furthermore, augmented cholesterol concentrations in the mitochondria have been detected in several diseases, such as cancers, myocardial ischemia, aging, steatohepatitis and Alzheimer’s disease [52]. Montero and colleagues have reported that the susceptibility of human hepatocellular carcinoma cells (HCCs) to chemotherapy signaling through mitochondria is increased after cholesterol depletion. In contrast, isolated mitochondria with increased cholesterol concentrations from HCCs are less sensitive to mitochondrial membrane permeabilization and release of Smac/DIABLO (an inhibitor of XIAP) or cytochrome *c* (an activator of caspase-9) in response to apoptotic stimuli [53]. In the current study, although we did not determine the cholesterol levels in the mitochondria, we reported the similar observations in the plasma membrane that the decrease of plasma membrane cholesterol intensified the susceptibility to cell death program in NSCLC when exposed to *para*-toluenesulfonamide.

Epidermal growth factor receptor (EGFR) has been intensively demonstrated to play a key role in the growth and survival of NSCLC. EGFR activity orchestrates multiple cellular processes responsible to tumor growth and progression, such as proliferation, metastasis, invasion and angiogenesis. Gefitinib is an orally active EGFR inhibitor that blocks EGFR tyrosine kinase through the binding to ATP binding site of the enzyme. Several clinical studies have shown that gefitinib monotherapy in NSCLC patients has moderate antitumor activity with good tolerability and have encouraged the use of gefitinib in combination with the agents of distinct mechanism [54]. Our data showed that the combinatory treatment between *para*-toluenesulfonamide and gefitinib in A549 and NCI-H460 cells synergistically induced apoptotic cell death.

In summary, the data suggest that *para*-toluenesulfonamide is an effective anticancer agent against NSCLC through G1 arrest of the cell cycle and subsequent cell apoptosis. Moreover, *para*-toluenesulfonamide also induces autophagy in NSCLC. The *para*-toluenesulfonamide action is attributed to the translational inhibition through the suppression of Akt/mTOR/p70S6K signaling pathways. Furthermore, disturbance of membrane cholesterol levels may play a crucial role in *para*-toluenesulfonamide action. In comparison with our previous report in CRPC, the current study demonstrates the novel finding of *para*-toluenesulfonamide-induced anticancer activity in PI3K-mutant and KRAS-mutant NSCLC cells. *Para*-toluenesulfonamide significantly decreases the levels of plasma membrane cholesterol in both cell lines. The autophagic activation and lysosomal degradation pathway may also play a role to *para*-toluenesulfonamide action. Moreover, *para*-toluenesulfonamide can dramatically sensitize gefitinib-mediated cell death in both NSCLC cell lines.

## 4. Materials and Methods

### 4.1. Materials

Human NSCLC cell lines, NCI-H460 and A549, were obtained from American Type Culture Collection (Rockville, MD, USA). RPMI 1640 medium, fetal bovine serum (FBS), penicillin and streptomycin were purchased from GIBCO/BRL Life Technologies (Grand Island, NY, USA). Antibodies of cyclin E, cyclin A, cyclin B, cyclin-dependent kinase (Cdk) 4, Cdk2, Cdk1, GAPDH, caveolin-1, LC3 and p62 were obtained from Santa Cruz Biotechnology, Inc. (Santa Cruz, CA, USA). Antibodies of Akt, p-Akt^Ser473^, cyclin D1, mTOR, p-mTOR^Ser2448^, 4E-BP1, p-4E-BP1^Thr37/46^ and p-p70S6K^Thr389^ were from Cell Signaling Technologies (Boston, MA, USA). P70S6K was from Abcam (Cambridge, UK). Carboxyfluorescein succinimidyl ester (CFSE) was from Molecular Probes Inc. (Eugene, OR, USA). Anti-mouse and anti-rabbit IgGs were from Jackson ImmunoResearch Laboratories, Inc. (West Grove, PA, USA). *Para*-toluenesulfonamide, sulforhodamine B (SRB), leupeptin, NaF, NaVO_4_, dithiothreitol, phenylmethylsulfonylfluoride (PMSF), trichloroacetic acid (TCA), water-soluble cholesterol (cholesterol-methyl-β-cyclodextrin), propidium iodide (PI) and all other chemical compounds were purchased from Sigma-Aldrich (St. Louis, MO, USA).

### 4.2. Cell Culture

NCI-H460 and A549 cells were cultured in RPMI 1640 medium with 10% FBS (*v*/*v*), penicillin (100 units/mL) and streptomycin (100 μg/mL) (all from GIBCO/BRL Life Technologies, Grand Island, NY, USA). Cells were cultivated at 37 °C in a humidified atmosphere containing 5% CO_2_.

### 4.3. SRB Assay

The cells were fixed with 10% TCA which acted as the population at initial time of compound treatment (TZ). After a 48-h treatment with vehicle or the agent, the cells were fixed (10% TCA) and stained with SRB at 0.4% (*w*/*v*) in 1% acetic acid. The cells were washed with 1% acetic acid, solubilized with 10 mM Tris and read on a microplate reader at 515 nm.

### 4.4. Colony Formation Assay

The experiment of anchorage-dependent clonogenic effect was performed. The cells were incubated in the absence or presence of the indicated agent for 10 days. The cell colonies were treated with 0.4% (*w*/*v*) crystal violet/20% methanol, lysed with 50 mM sodium citrate/50% ethanol and read on a microplate reader at 595 nm.

### 4.5. CFSE Staining Assay

The cells were incubated with 10 μM carboxyfluorescein succinimidyl ester (CFSE, Molecular Probes Inc., Eugene, OR, USA) at 37 °C for 10 min and then incubated in ice-cold 10% FBS-containing medium for 5 min. The cells were washed, centrifuged and seeded in 10% FBS-containing medium in the absence or presence of the agent for the indicated times at 37 °C. Cell proliferation was examined through detecting the reduction of fluorescence intensity in next-generation cells. Proliferation index was determined through calculating total number of divisions by the cell number that have undergone any division. MODFIT LT Version 3.3 (https://modfit-lt.software.informer.com/3.3 (accessed on 20 July 2011)) was used to determine the proliferation index and cell populations of parent or different generations.

### 4.6. Detection of Cell Population in Cell Cycle Progression

Cells were obtained and treated with ice-cold 70% alcohol for 30 min. The cells were washed with phosphate-buffered saline (PBS), centrifuged and incubated in phosphate-citric acid buffer (pH 7.8) for 30 min. After centrifugation, the cells were suspended with propidium iodide (PI, 80 μg/mL) solution with Triton X-100 (0.1% *v*/*v*) and RNase (100 μg/mL). DNA levels were examined using FACScan flow cytometric analysis.

### 4.7. Detection of Nucleosomal DNA Cleavage

Nucleosomal DNA cleavage was examined using commercial kit (Roche, Mannheim, Germany) to detect cytosol histone-associated cleaved DNA in cells after the induction of cell death. The cells were obtained and treated with lysis buffer. After centrifugation, supernatant part was obtained and incubated with HRP-conjugated anti-DNA-peroxidase antibody, following by washing and incubating with substrates of the antibody according to the manufacturer’s protocol. The plate was read by an ELISA reader (405 nm) to get the absorbance density values.

### 4.8. Western Blot Analysis

After the indicated exposure to the compound, the cells were obtained and treated with lysis solution. The protein concentrations were determined, treated with sample buffer at 90 °C for several minutes. Thirty micrograms of protein were electrophoresed in SDS-PAGE. The protein was identified using indicated antibody and secondary antibody (Jackson ImmunoResearch Laboratories, Inc., West Grove, PA, USA) and was determined using commercial chemiluminescence detection kit (Amersham, Buckinghamshire, UK). Protein expressions were quantified using Image Lab Software 6.0 (BIO-RAD) https://www.bio-rad.com/webroot/web/pdf/lsr/literature/Image-Lab-Software-v6.0.1-Release-Notes.pdf (accessed on 1 December 2017) and the protein expression relative to the respective control has been clarified in the legends.

### 4.9. Immunofluorescence Staining of Lysosomes and Cholesterol

Cells were live-stained with 50 nM LYSOTRACKER-RED DND-99 dye (Invitrogen, Grand Island, NY, USA) in medium at 37 °C for 30 min. Then, immunofluorescence staining was used to detect cholesterol localization (Cell-based cholesterol assay kit, Biovision, Milpitas, CA, USA). Culture medium was removed from the wells and the cells were fixed by Fixative Solution provided in the assay kit for 10 min and washed with the assay buffer for three times. The diluted Filipin III solution was added to each well and incubated in the dark for 45 min. The cells were gently washed with the assay buffer for two times. Then, the cells were analyzed by a confocal laser microscopic system (ZEISS LSM880).

### 4.10. Detection of Total Cellular Cholesterol Levels

The cells were washed for 3 times with cold PBS and were extracted with 200 μL chloroform: isopropanol: NP-40 (7:11:0.1) in a micro-homogenizer, centrifuged for 10 min at 15,000× *g*. The liquid (organic phase) was transferred to a new tube and was air dried at 50 °C to remove the chloroform. The samples were under vacuum for 30 min to remove the trace amounts of organic solvent. The dried lipids were sonicated and dissolved in 200 μL of 1X Assay Diluent using the commercial TOTAL CHOLESTEROL ASSAY Kit (Colorimetric, Cell Biolabs, San Diego, CA, USA). The cholesterol levels were examined according to the manual. The plate was read immediately at the 540–570 nm range. The concentration of cholesterol was calculated by comparing the sample absorbance values to the cholesterol standard curve.

### 4.11. Purification of Plasma Membrane

Cells (5 × 10^8^) were collected and re-suspend the cell pellet in 1 mL of ice-cold Homogenization Buffer Solution (0.25 M sucrose, 1 mM EDTA, 1mM MgCl_2_, 20 mM Hepes-NaOH, pH 7.4). Then, the cells were homogenized by sonication or a pre-chilled Dounce Homogenizer. After efficient homogenization, the samples were centrifuged at 700× *g* for 10 min at 4 °C and carefully removed and the fatty residue was discarded from the top of the Supernatant. Supernatant was collected and sonicated by a microsonicator on ice. The supernatant (1 mL) was mixed with 4 mL of the 50% OPTIPREP (Sigma-Aldrich, D1556). The diluted samples were overlaid with 6.3 mL 25% OPTIPREP and 1.4 mL 2.5% OPTIPREP, and centrifuged at 200,000× *g* for 90 min at 4 °C using a Beckman type SW41Ti rotor (Beckman Instruments, Palo Alto, CA, USA). At the end of the ultracentrifugation, the plasma membrane fraction was in the visible band at the interface of the 2.5%/25% gradient solution.

### 4.12. Data Analysis

Data are expressed as mean ± SEM of at least three independent experiments. Computerized image analysis system Lab^TM^ Software (Bio-Rad Laboratories, Hercules, CA, USA) was adopted to quantify experimental results of Western blot analysis. One-way ANOVA followed by a Newman–Keuls post hoc test is applied. *p*-values less than 0.05 are considered statistically significant.

## Supplementary Materials

The following are available online, Figure S1: Effect of *para*-toluenesulfonamide on the inhibition of cell proliferation, Figure S2: Effect of *para*-toluenesulfonamide on the protein expression of several kinases, Figure S3: Effect of several kinase inhibitors on the protein expression of several proteins in their total forms or phosphorylated forms, Figure S4: Effect of autophagy inhibitors on several protein expressions, Figure S5: Effect of autophagy inhibitors on *para*-toluenesulfonamide-induced sub-G1 population and cell death, Figure S6: Effect of combination treatment on cell death in A549 and NCI-H460 cells.

## Sample Availability

Samples of the compounds are not available from the authors.

## Abbreviations

Cdk	Cyclin-dependent kinase
CFSE	Carboxyfluorescein succinimidyl ester
4E-BP1	eIF4E-binding protein 1
EGFR	Epidermal growth factor receptor
eIF4E	Eukaryotic translation initiation factor 4E
FBS	Fetal bovine serum
MAPK	Mitogen-activated protein kinase
mTOR	Mammalian target of rapamycin
NSCLC	Non-small-cell lung cancer
p70S6K	p70S6 kinase
PBS	Phosphate-buffered saline
PI	Propidium iodide
PI3K	Phosphatidylinositol 3-kinase
PMSF	Phenylmethylsulfonylfluoride
SRB	Sulforhodamine B
TCA	Trichloroacetic acid
TCGA	The Cancer Genome Atlas

## References

[1] Testa U., Castelli G., Pelosi E. (2018). Lung cancers: Molecular characterization, clonal heterogeneity and evolution, and cancer stem cells. Cancers.

[2] Bartsch H., Dally H., Popanda O., Risch A., Schmezer P. (2007). Genetic risk profiles for cancer susceptibility and therapy response. Recent Results Cancer Res..

[3] Di Maio M., Gridelli C., Gallo C., Shepherd F., Piantedosi F.V., Cigolari S., Manzione L., Illiano A., Barbera S., Robbiati S.F. (2005). Chemotherapy-induced neutropenia and treatment efficacy in advanced non-small-cell lung cancer: A pooled analysis of three randomized trials. Lancet Oncol..

[4] Ribaudo G., Zanforlin E., Zagotto G. (2018). Overcoming resistance in non-small-cell lung cancer: A practical lesson for the medicinal chemist. Arch. Pharm..

[5] Pakkala S., Ramalingam S.S. (2018). Personalized therapy for lung cancer: Striking a moving target. JCI Insight.

[6] Mollinedo F., Gajate C. (2015). Lipid rafts as major platforms for signaling regulation in cancer. Adv. Biol. Regul..

[7] Hu J., La Vecchia C., de Groh M., Negri E., Morrison H., Mery L., Canadian Cancer Registries Epidemiology Research Group (2012). Dietary cholesterol intake and cancer. Ann. Oncol..

[8] Ahmad F., Sun Q., Patel D., Stommel J.M. (2019). Cholesterol metabolism: A potential therapeutic target in glioblastoma. Cancers.

[9] Kuzu O.F., Noory M.A., Robertson G.P. (2016). The role of cholesterol in cancer. Cancer Res..

[10] Brusselmans K., Timmermans L., Van de Sande T., Van Veldhoven P.P., Guan G., Shechter I., Claessens F., Verhoeven G., Swinnen J.V. (2007). Squalene synthase, a determinant of Raft-associated cholesterol and modulator of cancer cell proliferation. J. Biol. Chem..

[11] Yang Y.F., Jan Y.H., Liu Y.P., Yang C.J., Su C.Y., Chang Y.C., Lai T.C., Chiou J., Tsai H.Y., Lu J. (2014). Squalene synthase induces tumor necrosis factor receptor 1 enrichment in lipid rafts to promote lung cancer metastasis. Am. J. Respir. Crit. Care Med..

[12] Hanai J., Doro N., Sasaki A.T., Kobayashi S., Cantley L.C., Seth P., Sukhatme V.P. (2012). Inhibition of lung cancer growth: ATP citrate lyase knockdown and statin treatment leads to dual blockade of mitogen-activated protein kinase (MAPK) and phosphatidylinositol-3-kinase (PI3K)/AKT pathways. J. Cell Physiol..

[13] Gao Y., Gao Y., Guan W., Huang L., Xu X., Zhang C., Chen X., Wu Y., Zeng G., Zhong N. (2013). Antitumor effect of para-toluenesulfonamide against lung cancer xenograft in a mouse model. J. Thorac. Dis..

[14] Liu Z., Liang C., Zhang Z., Pan J., Xia H., Zhong N., Li L. (2015). Para-toluenesulfonamide induces tongue squamous cell carcinoma cell death through disturbing lysosomal stability. Anticancer Drugs.

[15] Hsu J.L., Leu W.J., Hsu L.C., Liu S.P., Zhong N.S., Guh J.H. (2018). Para-toluenesulfonamide induces anti-tumor activity through Akt-dependent and –independent mTOR/p70S6K pathway: Roles of lipid raft and cholesterol contents. Front. Pharmacol..

[16] He Q., Kuang A.R., Guan Y.S., Liu Y.Q. (2012). Puncture injection of para-toluenesulfonamide combined with chemoembolization for advanced hepatocellular carcinoma. World J. Gastroenterol..

[17] He J., Ying W., Yang H., Xu X., Shao W., Guan Y., Jiang M., Wu Y., Zhong B., Wang D. (2009). Gemcitabine plus cisplatin chemotherapy with concurrent para-toluenesulfonamide localinjection therapy for peripherally advanced nonsmall cell lung cancer larger than 3 cm in the greatest dimension. Anticancer Drugs.

[18] Guan W.J., Li S.Y., Zhong N.S. (2018). Effects of para-toluenesulfonamide intratumoral injection on pulmonary adenoid cystic carcinoma complicating with severe central airway obstruction: A 5-year follow-up study. J. Thorac. Dis..

[19] Li S.Y., Li Q., Guan W.J., Huang J., Yang H.P., Wu G.M., Jin F.G., Hu C.P., Chen L.A., Xu G.L. (2016). Effects of para-toluenesulfonamide intratumoral injection on non-small cell lung carcinoma with severe central airway obstruction: A multi-center, non-randomized, single-arm, open-label trial. Lung Cancer.

[20] Qin X., Jiang B., Zhang Y. (2016). 4E-BP1, a multifactor regulated multifunctional protein. Cell Cycle.

[21] Wu W., Hu W., Han W.B., Liu Y.L., Tu Y., Yang H.M., Fang Q.J., Zhou M.Y., Wan Z.Y., Tang R.M. (2018). Inhibition of Akt/mTOR/p70S6K signaling activity with Huangkui capsule alleviates the early glomerular pathological changes in diabetic nephropathy. Front. Pharmacol..

[22] Gao B., Roux P.P. (2015). Translational control by oncogenic signaling pathways. Biochim. Biophys. Acta..

[23] Ghayad S.E., Cohen P.A. (2010). Inhibitors of the PI3K/Akt/mTOR pathway: New hope for breast cancer patients. Recent Pat. Anticancer Drug Discov..

[24] Silvius J.R. (2003). Role of cholesterol in lipid raft formation: Lessons from lipid model systems. Biochim. Biophys. Acta.

[25] Zhuang L., Lin J., Lu M.L., Solomon K.R., Freeman M.R. (2002). Cholesterol-rich lipid rafts mediate akt-regulated survival in prostate cancer cells. Cancer Res..

[26] Calay D., Vind-Kezunovic D., Frankart A., Lambert S., Poumay Y., Gniadecki R.J. (2010). Inhibition of Akt signaling by exclusion from lipid rafts in normal and transformed epidermal keratinocytes. J. Investig. Dermatol..

[27] Cheng J., Ohsaki Y., Tauchi-Sato K., Fujita A., Fujimoto T. (2006). Cholesterol depletion induces autophagy. Biochem. Biophys. Res. Commun..

[28] Motoyama K., Kameyama K., Onodera R., Araki N., Hirayama F., Uekama K., Arima H. (2009). Involvement of PI3K-Akt-Bad pathway in apoptosis induced by 2,6-di-O-methyl-beta-cyclodextrin, not 2,6-di-O-methyl-alpha-cyclodextrin, through cholesterol depletion from lipid rafts on plasma membranes in cells. Eur. J. Pharm. Sci..

[29] Chou T.C. (2010). Drug combination studies and their synergy quantification using the Chou-Talalay method. Cancer Res..

[30] Lin X., Liu L., Fu Y., Gao J., He Y., Wu Y., Lian X. (2018). Dietary cholesterol intake and risk of lung cancer: A meta-analysis. Nutrients.

[31] Weinstein J.N., Collisson E.A., Mills G.B., Shaw K.R., Ozenberger B.A., Ellrott K., Shmulevich I., Sander C., Stuart J.M., Cancer Genome Atlas Research Network (2013). The Cancer Genome Atlas Pan-Cancer analysis project. Nat. Genet..

[32] Henslee A.B., Steele T.A. (2018). Combination statin and chemotherapy inhibits proliferation and cytotoxicity of an aggressive natural killer cell leukemia. Biomark. Res..

[33] Mandal C.C., Rahman M.M. (2014). Targeting intracellular cholesterol is a novel therapeutic strategy for cancer treatment. J. Cancer Sci. Ther..

[34] Roskoski R. (2019). Cyclin-dependent protein serine/threonine kinase inhibitors as anticancer drugs. Pharmacol. Res..

[35] Qiu Z.X., Zhang K., Qiu X.S., Zhou M., Li W.M. (2013). The prognostic value of phosphorylated AKT expression in non-small cell lung cancer: A meta-analysis. PLoS ONE.

[36] Gately K., Al-Alao B., Dhillon T., Mauri F., Cuffe S., Seckl M., O’Byrne K. (2012). Overexpression of the mammalian target of rapamycin (mTOR) and angioinvasion are poor prognostic factors in early stage NSCLC: A verification study. Lung Cancer.

[37] Liu D., Huang Y., Chen B., Zeng J., Guo N., Zhang S., Liu L., Xu H., Mo X., Li W. (2011). Activation of mammalian target of rapamycin pathway confers adverse outcome in nonsmall cell lung carcinoma. Cancer.

[38] Ayuso M.I., Hernández-Jiménez M., Martín M.E., Salinas M., Alcázar A. (2010). New hierarchical phosphorylation pathway of the translational repressor eIF4E-bindingprotein 1 (4E-BP1) in ischemia-reperfusion stress. J. Biol. Chem..

[39] Hinnebusch A.G. (2012). Translational homeostasis via eIF4E and 4E-BP1. Mol. Cell..

[40] Shen W., Boyle D.W., Liechty E.A. (2005). Changes in 4E-BP1 and p70S6K phosphorylation in skeletal muscle of the ovine fetus after prolonged maternal fasting: Effects of insulin and IGF-I. Pediatr. Res..

[41] Zeng J., Zhang H., Tan Y., Sun C., Liang Y., Yu J., Zou H. (2018). Aggregation of lipid rafts activates c-met and c-Src in non-small cell lung cancer cells. BMC Cancer.

[42] Head B.P., Patel H.H., Insel P.A. (2014). Interaction of membrane/lipid rafts with the cytoskeleton: Impact on signaling and function: Membrane/lipid rafts, mediators of cytoskeletal arrangement and cell signaling. Biochim. Biophys. Acta.

[43] Gao X., Lowry P.R., Zhou X., Depry C., Wei Z., Wong G.W., Zhang J. (2011). PI3K/Akt signaling requires spatial compartmentalization in plasma membrane microdomains. Proc. Natl. Acad. Sci. USA.

[44] Gajate C., Mollinedo F. (2007). Edelfosine and perifosine induce selective apoptosis in multiple myeloma by recruitment of death receptors and downstream signaling molecules into lipid rafts. Blood.

[45] Lima R.T., Sousa D., Gomes A.S., Mendes N., Matthiesen R., Pedro M., Marques F., Pinto M.M., Sousa E., Vasconcelos M.H. (2018). The antitumor activity of a lead thioxanthone is associated with alterations in cholesterol localization. Molecules.

[46] Zoncu R., Efeyan A., Sabatini D.M. (2011). mTOR: From growth signal integration to cancer, diabetes and ageing. Nat. Rev. Mol. Cell. Biol..

[47] Mariño G., Niso-Santano M., Baehrecke E.H., Kroemer G. (2014). Self-consumption: The interplay of autophagy and apoptosis. Nat. Rev. Mol. Cell. Biol..

[48] Eid W., Dauner K., Courtney K.C., Gagnon A., Parks R.J., Sorisky A., Zha X. (2017). mTORC1 activates SREBP-2 by suppressing cholesterol trafficking to lysosomes in mammalian cells. Proc. Natl. Acad. Sci. USA.

[49] Zhang Y., Chen X., Gueydan C., Han J. (2018). Plasma membrane changes during programmed cell deaths. Cell Res..

[50] Duarte A., Poderoso C., Cooke M., Soria G., Cornejo Maciel F., Gottifredi V., Podestá E.J. (2012). Mitochondrial fusion is essential for steroid biosynthesis. PLoS ONE.

[51] Hall P.F., Almahbobi G. (1997). Roles of microfilaments and intermediate filaments in adrenal steroidogenesis. Microsc. Res. Tech..

[52] Martin L.A., Kennedy B.E., Karten B.J. (2016). Mitochondrial cholesterol: Mechanisms of import and effects on mitochondrial function. Bioenerg. Biomembr..

[53] Montero J., Morales A., Llacuna L., Lluis J.M., Terrones O., Basañez G., Antonsson B., Prieto J., García-Ruiz C., Colell A. (2008). Mitochondrial cholesterol contributes to chemotherapy resistance in hepatocellular carcinoma. Cancer Res..

[54] Giaccone G., Herbst R.S., Manegold C., Scagliotti G., Rosell R., Miller V., Natale R.B., Schiller J.H., Von Pawel J., Pluzanska A. (2004). Gefitinib in combination with gemcitabine and cisplatin in advanced non-small-cell lung cancer: A phase III trial--INTACT 1. J. Clin. Oncol..

