# Supplementary figures and images for: Autophagic Activation and Decrease of Plasma Membrane Cholesterol Contribute to Anticancer Activities in Non-Small Cell Lung Cancer
Source: Molecules. 2021 Oct 1;26(19):5967. doi: 10.3390/molecules26195967 (PMC8512437; doi:10.3390/molecules26195967)

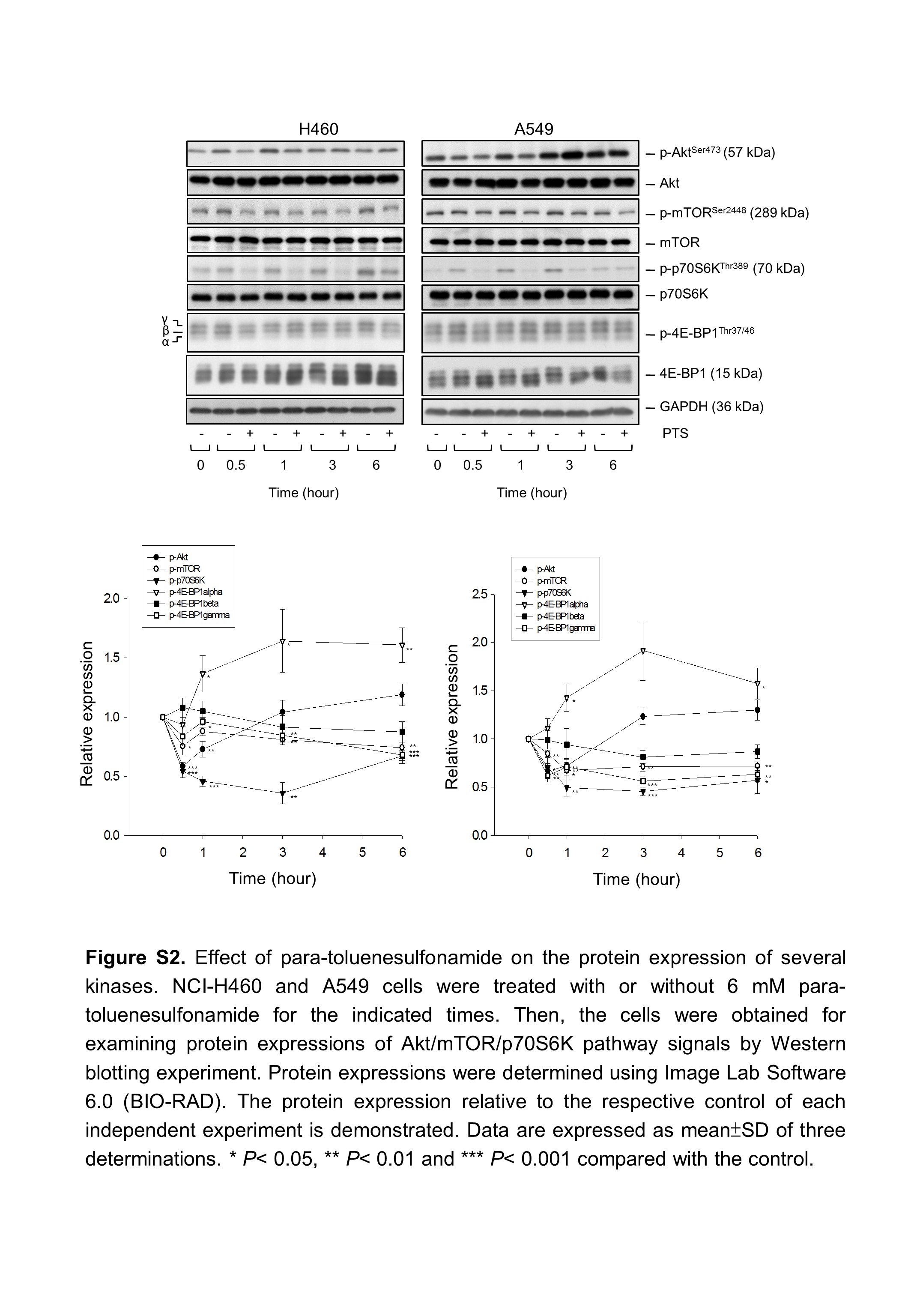

Supplement: Supplementary file 1 [file molecules-26-05967-s001.zip › Supplementary Figure 2.tif]

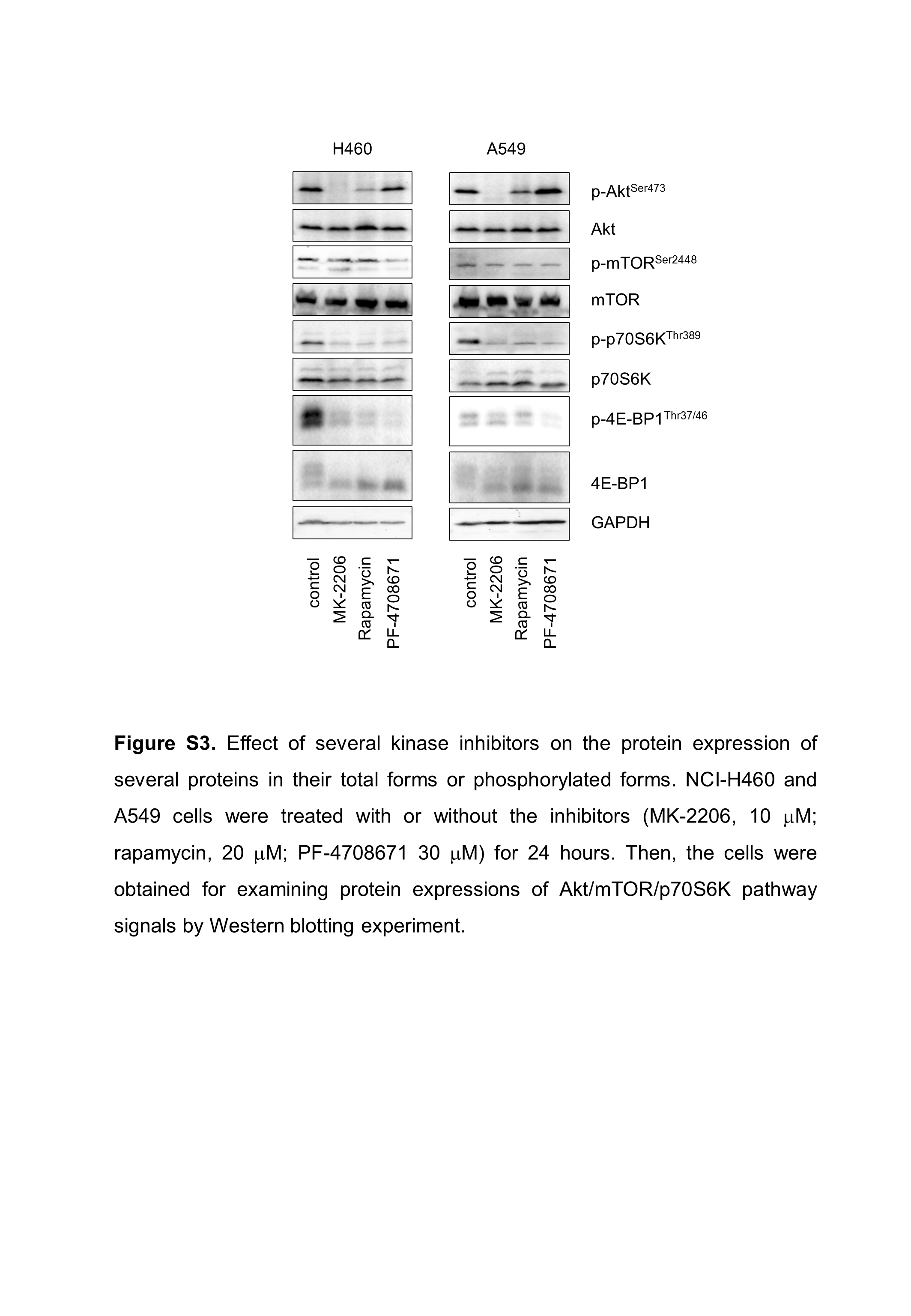

Supplement: Supplementary file 1 [file molecules-26-05967-s001.zip › Supplementary Figure 3.tif]

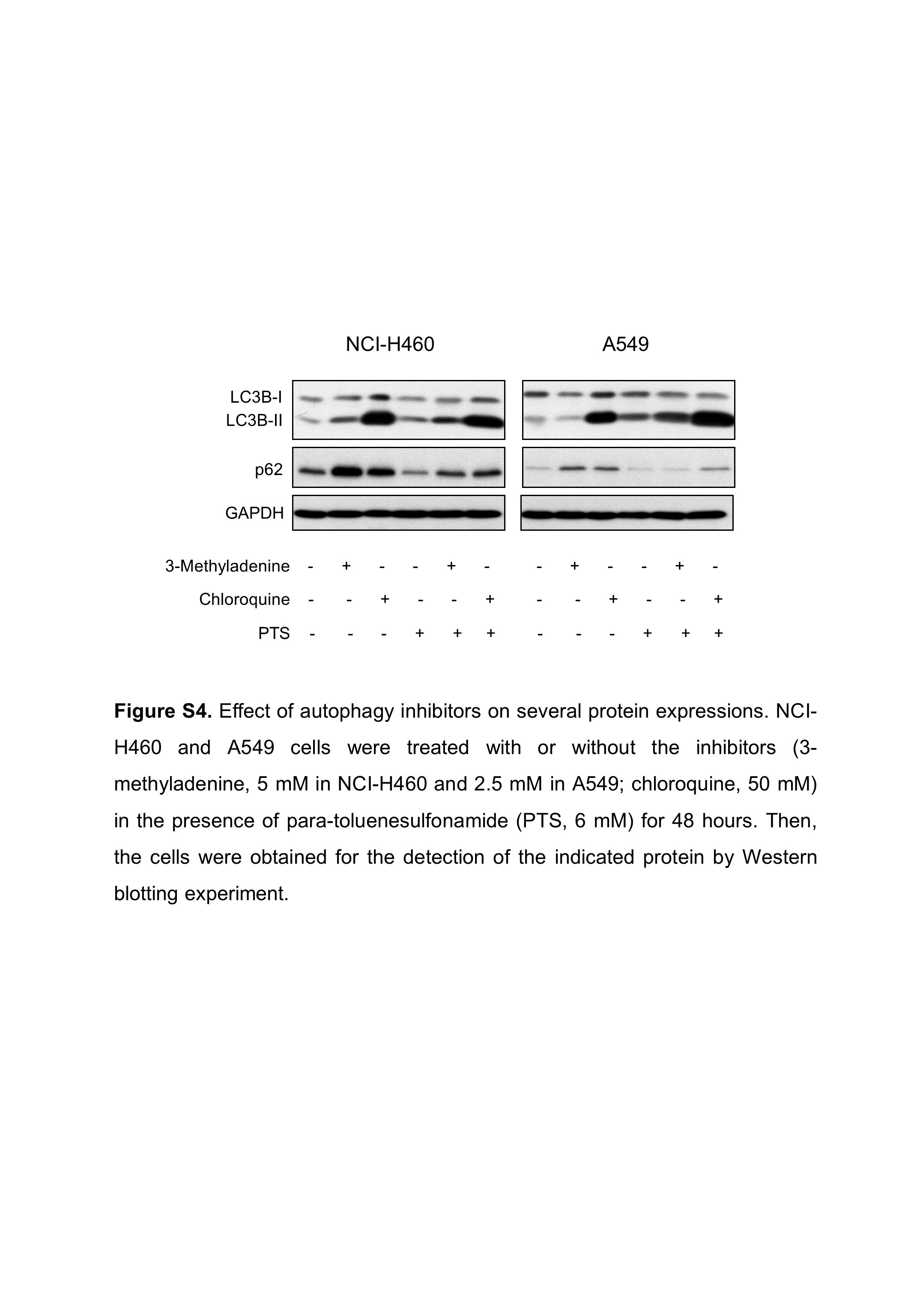

Supplement: Supplementary file 1 [file molecules-26-05967-s001.zip › Supplementary Figure 4.tif]

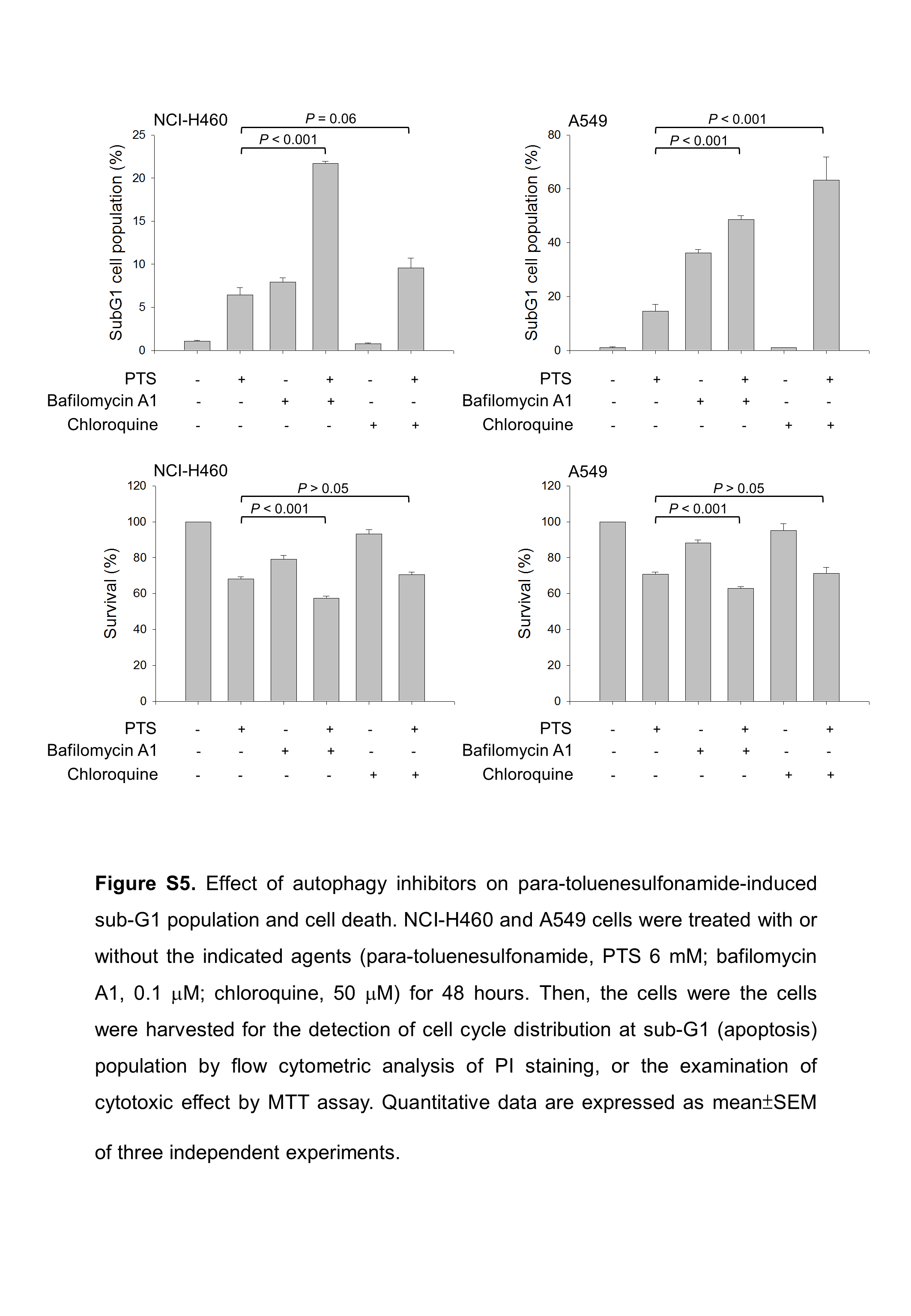

Supplement: Supplementary file 1 [file molecules-26-05967-s001.zip › Supplementary Figure 5.tif]

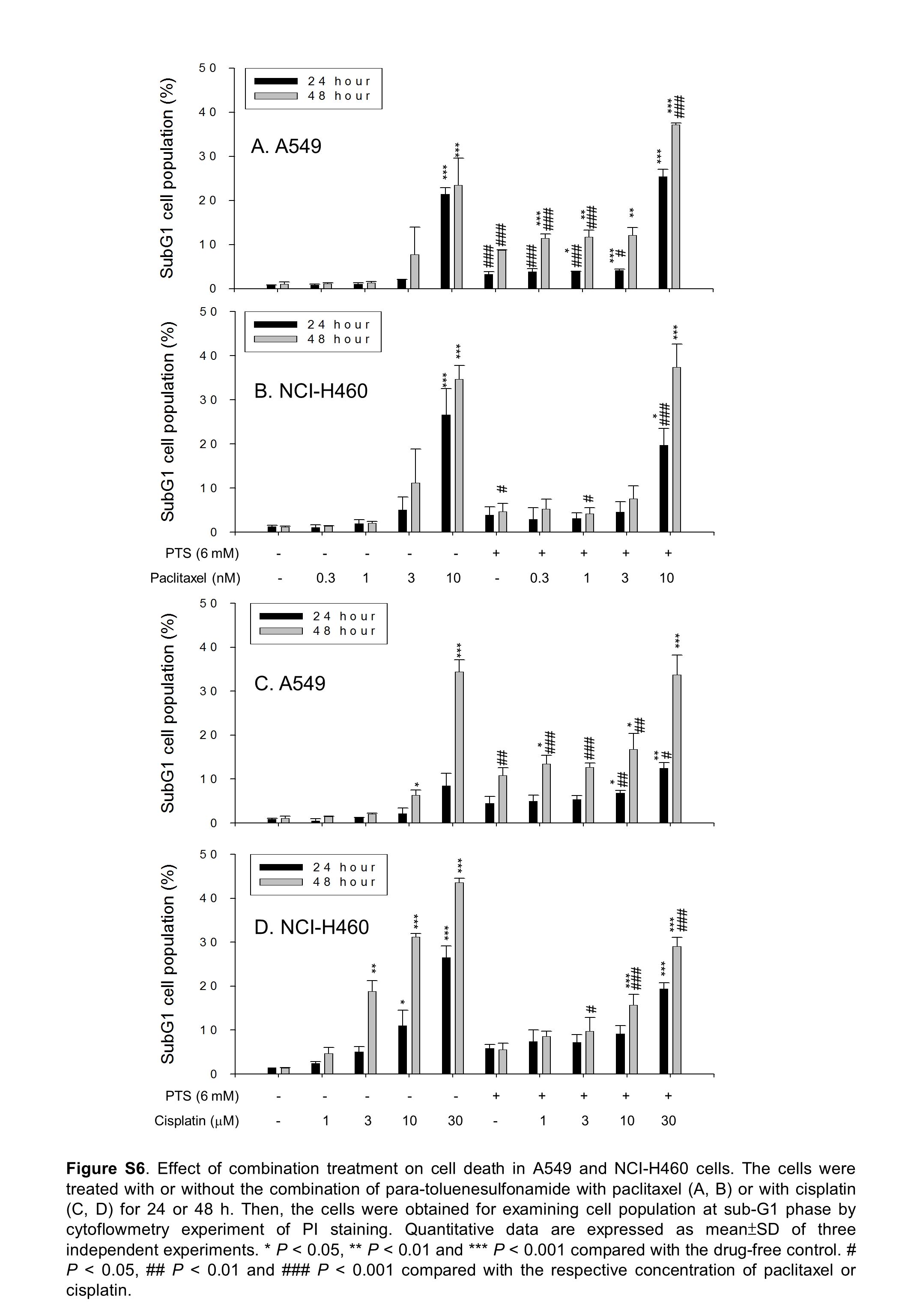

Supplement: Supplementary file 1 [file molecules-26-05967-s001.zip › Supplementary Figure 6.tif]

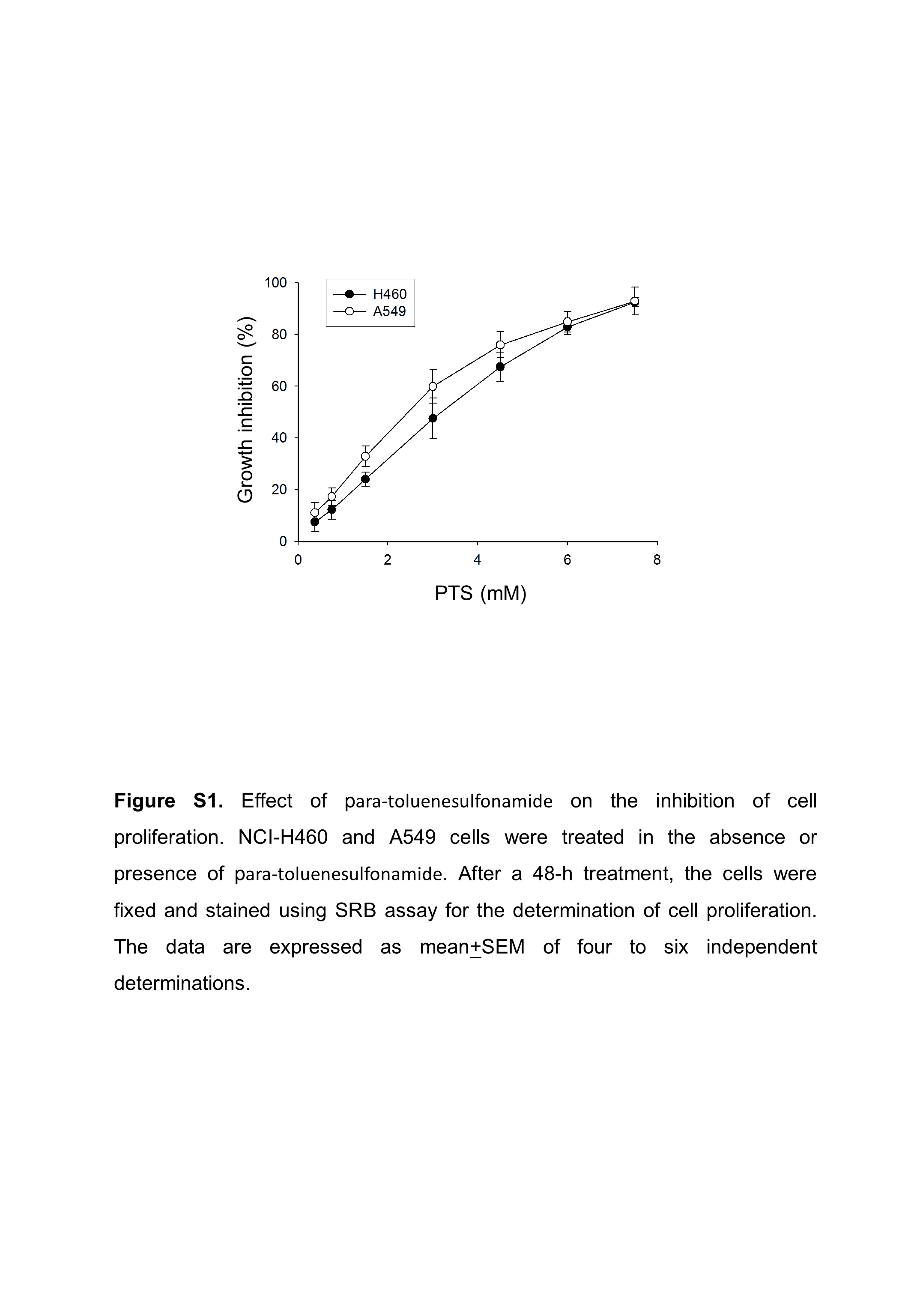

Supplement: Supplementary file 1 [file molecules-26-05967-s001.zip › Supplementary Figure 1.tif]
